# Comparison of the Genetic Features Involved in *Bacillus subtilis* Biofilm Formation Using Multi-Culturing Approaches

**DOI:** 10.3390/microorganisms9030633

**Published:** 2021-03-18

**Authors:** Yasmine Dergham, Pilar Sanchez-Vizuete, Dominique Le Coq, Julien Deschamps, Arnaud Bridier, Kassem Hamze, Romain Briandet

**Affiliations:** 1 Micalis Institute, INRAE, AgroParisTech, Université Paris-Saclay, 78350 Jouy-en-Josas, France; yasmin.dorghamova-dergham@inrae.fr (Y.D.); pilarsanviz@gmail.com (P.S.-V.); dominique.le-coq@inrae.fr (D.L.C.); julien.deschamps@inrae.fr (J.D.); 2 Faculty of Science, Lebanese University, 1003 Beirut, Lebanon; kassem.hamze@ul.edu.lb; 3 Centre National de la Recherche Scientifique (CNRS), Micalis Institute, INRAE, AgroParisTech, Université Paris-Saclay, 78350 Jouy-en-Josas, France; 4 Fougères Laboratory, Antibiotics, Biocides, Residues and Resistance Unit, Anses, 35300 Fougères, France; arnaud.bridier@anses.fr

**Keywords:** *Bacillus subtilis*, NDmed, biofilm, pellicle, complex macrocolonies, swarming, confocal laser scanning microscopy (CLSM)

## Abstract

Surface-associated multicellular assemblage is an important bacterial trait to withstand harsh environmental conditions. *Bacillus subtilis* is one of the most studied Gram-positive bacteria, serving as a model for the study of genetic pathways involved in the different steps of 3D biofilm formation. *B. subtilis* biofilm studies have mainly focused on pellicle formation at the air-liquid interface or complex macrocolonies formed on nutritive agar. However, only few studies focus on the genetic features of *B. subtilis* submerged biofilm formation and their link with other multicellular models at the air interface. NDmed, an undomesticated *B. subtilis* strain isolated from a hospital, has demonstrated the ability to produce highly structured immersed biofilms when compared to strains classically used for studying *B. subtilis* biofilms. In this contribution, we have conducted a multi-culturing comparison (between macrocolony, swarming, pellicle, and submerged biofilm) of *B. subtilis* multicellular communities using the NDmed strain and mutated derivatives for genes shown to be required for motility and biofilm formation in pellicle and macrocolony models. For the 15 mutated NDmed strains studied, all showed an altered phenotype for at least one of the different culture laboratory assays. Mutation of genes involved in matrix production (i.e., *tasA*, *epsA-O*, *cap*, *ypqP*) caused a negative impact on all biofilm phenotypes but favored swarming motility on semi-solid surfaces. Mutation of *bslA*, a gene coding for an amphiphilic protein, affected the stability of the pellicle at the air-liquid interface with no impact on the submerged biofilm model. Moreover, mutation of *lytF,* an autolysin gene required for cell separation, had a greater effect on the submerged biofilm model than that formed at aerial level, opposite to the observation for *lytABC* mutant. In addition, *B. subtilis* NDmed with *sinR* mutation formed wrinkled macrocolony, less than that formed by the wild type, but was unable to form neither thick pellicle nor structured submerged biofilm. The results are discussed in terms of the relevancy to determine whether genes involved in colony and pellicle formation also govern submerged biofilm formation, by regarding the specificities in each model.

## 1. Introduction

Bacteria in nature frequently exist in communities that display complex social behavior, which involves intercellular signaling to permit survival and dissemination in a wide variety of habitats [1]. Even within a pure culture biofilm, where cells are genetically identical, different patterns of gene expression co-exist and therefore produce subpopulations of functionally distinct cell types [2]. Surface-associated biofilm develops in a sequential process in which sessile bacterial cells secrete extracellular matrix and aggregate as structured multicellular groups [3,4]. In nature, microbial biofilms participate in many biogeochemical cycling processes for most elements in water, soil, sediments, and subsurface environments [5]. In addition, utilization of microbial antagonists as biological control agents is a promising biotechnological alternative to the use of pesticides, which often accumulate in plants and end up by affecting humans in a direct or indirect way [6]. However, in terms of public health and with the medical science progress, more and more medical devices and/or artificial organs are being applied in the treatment of human diseases. As a consequence, biofilm-associated infections has become also frequent. It has been estimated that many bacterial infections in human are correlated with biofilm formation and are associated with the indwelling medical devices (such as catheters or needles) [7].

Over the last decades, *Bacillus subtilis*, a Gram-positive, motile, spore-forming bacterium has served as a model organism for molecular studies on biofilm formation [5]. These studies were mainly based on the development of complex macrocolonies on the agar-air interface, or floating pellicle at the air-liquid interface, and only few on submerged biofilms [8,9,10,11,12,13,14]. These models allowed highlighting that the transition from motile to sessile biofilm lifestyle, and vice versa, is controlled by complex genes regulatory networks. Four pairs of global regulators—the Spo0A/AbrB, SinI/SinR, SlrR/SlrA, and DegS/DegU—have been shown to play major roles, directly and indirectly, on both the formation and development of complex multicellular communities and on expression of the motility-involved genes [8,12,15,16,17,18,19,20]. Flagella required for motility are partly encoded by the *fla/che* operon, which, in addition to flagellar genes, includes chemotaxis genes and the *sigD* gene. In turn, the sigma D factor has been shown to direct transcription of other flagellar genes outside the *fla/che* operon (i.e., *hag* gene and other SigD-dependent motility genes) and genes involved in autolytic enzymes synthesis (*lytC, lytD*, and *lytF*) that mediate the separation of sister cells after cell division [21,22,23,24]. On the other hand, Spo0A phosphorylation represses two negative biofilm formation regulators, AbrB and SinR, therefore leading to expression of genes involved in the synthesis of biofilm matrix (polysaccharide synthesis by *epsA-O*, amyloid like fiber TasA encoded by the *tapA-sipW-tasA* operon, and the amphiphilic matrix protein produced by *bslA*) [2,25].

In specific conditions, cells from a bacterial colony can become highly motile and migrate over the substrate with specific collective patterns, a process known as swarming [4]. Swarming—a remarkable example of cooperative behavior in bacteria—is a mass, coordinated, and rapid migration (2 to 10 mm/hr) of cells on a surface [26]. In *B. subtilis*, this developmental process is observed on semi-solid agar (0.6%–1% agar) and has been shown to be completely dependent on flagella and surfactin production [26,27,28,29].

In 2001, Hamon and Lazazzera have shown that *B. subtilis* has the ability to adhere to abiotic surfaces and form structured biofilms [8], which have grabbed biofilm researches to reconsider the importance of the immersed surface-associated biofilm model for this species. In this context, architectural comparative submerged biofilm studies performed on various *B. subtilis* strains from different origins, including NCIB3610 and 168 reference strains, have revealed an undomesticated *B. subtilis* NDmed strain as able to form the highest submerged biofilm biovolume [11,13].

The NDmed strain, isolated from a hospital endoscope washer-disinfector was found to resist to the action of peracetic acid (an oxidizing agent commonly used in formulations used for the endoscope disinfection) and to have the ability to protect the pathogen *Staphylococcus aureus* in mixed biofilms [30,31]. By the use of confocal and electronic microscopy techniques, it has been shown that the hyper-resistant phenotype was related to the complex architectural biofilm formed and to the large amount of extracellular matrix produced that could prevent the diffusion-reaction of oxidizing agents [30]. Moreover, further genetic comparison between NDmed and other *B. subtilis* reference strains pinpointed that the *ypqP* gene (renamed *spsM* [32]), potentially involved in the synthesis of polysaccharide, was involved indirectly in this resistance by participating to the strong spatial organization of the *B. subtilis* NDmed biofilms, both at air and liquid interfaces [13]. This gene is disrupted by the SPβ prophage in both *B. subtilis* NCIB3610 and 168 strains [13]. These new observations suggested that interfaces between surfaces and liquids could, as for most other bacteria, be a relevant biotope for *B. subtilis* biofilm.

Our knowledge for the molecular mechanisms controlling the formation and the behavior of *B. subtilis* 3D communities is still limited. In this contribution, *B. subtilis* NCIB3610 and 168 strains were compared to NDmed in different laboratory culture conditions. Moreover, 15 mutants derived from the NDmed strain and defective in genes previously described as triggering biofilm formation were compared through a multi-culturing approach using four multicellular models, at the interface with air (solid agar, semi-solid agar, liquid medium) or at the interface between a solid surface (polystyrene) and a liquid medium, submerged model. Thus, this provided a global view over the different biofilm laboratory assays used to study the effect of gene mutation on both motility and biofilm formation in *B. subtilis* wild type.

## 2. Materials and Methods

### 2.1. Bacterial Strains and Growth Conditions

All bacterial strains and mutants used in this study are listed in Table 1. The *B. subtilis* NDmed derivatives mutated in various genes were obtained by transformation with chromosomal DNA extracted from strains carrying the corresponding different alleles of interest marked with a suitable antibiotic resistance cassette. Transforming chromosomal DNA was extracted according to the method of Marmur [33], and transformation of *B. subtilis* was performed according to the method of Anagnostopoulos and Spizizen [34], including the use of the MGI and MGII media of Borenstein and Ephrati-Elizur [35]. Transformants were selected on Lysogeny Broth (LB) plates supplemented with the relevant antibiotic at the following concentrations: spectinomycin, 100 µg ml^−1^; chloramphenicol, 4 µg ml^−1^; erythromycin, 0.5 µg ml^−1^; tetracycline, 10 µg ml^−1^; neomycin and kanamycin, 8 µg ml^−1^. Before each experiment, cells were subcultured in Tryptone Soya Broth (TSB, BioMérieux, France; pH 7.2) and supplemented with antibiotics when necessary. For biofilm formation, bacteria were grown in TSB at 30 °C for 8 h with agitation, then diluted 1/100 in 10 mL TSB incubated overnight at 30 °C. This culture was then used to grow biofilms on different assays. Bacteria for swarming experiments were grown with agitation at 37 °C in synthetic B-medium composed of (all final concentrations; pH 7.2) 15 mM (NH_4_)_2_SO_4_, 8 mM MgSO_4_.7H_2_O, 27 mM KCl, 7 mM sodium citrate.2H_2_O, 50 mM Tris/HCl (pH 7.5), and 2 mM CaCl_2_.2H_2_O, 1 μM FeSO_4_.7H_2_O, 10 μM MnSO_4_.4H_2_O, 0.6 mM KH_2_PO_4_, 4.5 mM glutamic acid (pH 8), 862 μM lysine, 784 μM tryptophan, 1 mM threonine and 0.5% glucose were added before use [36]. Antibiotics were added to bacterial cultures when needed.

**Table 1:** *Bacillus subtilis* strains used in this study.

Strain	Genotype or Isolation Source	Construction ^a^ or Reference
NDmed	Undomesticated, isolated from endoscope washer-disinfectors	[31]
NCIB3610	Natural isolate, less domesticated	[37]
168	*trpC2* (domesticated strain)	[37]
GM3248	NDmed Δ*ypqP*:: kan	[13]
GM3533	NDmed Δ*sinR*:: cm	Tf NDmed/DNA ABS840 [38]
GM3535	NDmed Δ*epsA-O*:: tet	Tf NDmed/DNA GM3532 [NCIB3610, Δ*tasA*:: kan, Δ*epsA-O::* tet] ( our lab collection)
GM3539	NDmed Δ*sinI*:: kan	Tf NDmed/DNA ABS803 [39]
GM3545	NDmed Δ*cap*:: pKPSd/ cm	Tf NDmed/DNA GM3543 [NCIB3610 Δ*cap*:: pKPSd/ cm] (our lab collection)
GM3555	NDmed Δ*abrB*:: cm	Tf NDmed/DNA MM1717 [40]
GM3559	NDmed Δ*degU*:: neo	Tf NDmed/DNA GM719 [41]
GM3561	NDmed Δ*bslA*:: cm	Tf NDmed/DNA NRS2097 [20]
GM3602	NDmed Δ*lytF*:: spec	Tf NDmed/DNA NRS3295 [42]
GM3611	NDmed Δ*lytABC*:: kan	Tf NDmed/DNA NRS3295 [42]
GM3614	NDmed Δ*tasA*:: kan	Tf NDmed/DNA GM3532 [NCIB3610, Δ*tasA*:: kan, Δ*epsA-O::* tet] ( our lab collection)
GM3618	NDmed Δ*slrR*:: spec	Tf NDmed/DNA GM3598 [NCIB3610 Δ*slrR::* spec] (our lab collection)
GM3619	NDmed Δ*srfAA*:: ery	Tf NDmed/DNA GM3599 [NCIB3610 Δ*srfAA*:: ery] (our lab collection)
GM3652	NDmed *amyE*:: Phyperspank-GFP/spec, Δ*hag*::cm	Tf NDmedGFP [30]/DNA OMG954 [29]
GM3671	NDmed *amyE*:: Phyperspank-GFP/spec, Δ*spo0A*:: Kan	Tf NDmedGFP [30]/DNA FBT2 [43]

^a^ TF NDmed/DNA stands for transformation of NDmed by chromosomal DNA of indicated strains.

### 2.2. Submerged Biofilm Developmental Assays

Submerged biofilms were grown on the surface of polystyrene 96-well microtiter plates with a µclear^®^ base (Greiner Bio-one, France) enabling high-resolution fluorescence imaging as previously described [44]. An amount of 200 µL of an overnight culture in TSB (adjusted to an OD 600 nm of 0.02) was added in each well. The microtiter plate was then incubated at 30 °C for 90 min to allow the bacteria to adhere to the bottom of the wells. Wells were then rinsed with TSB to eliminate non-adherent bacteria and refilled with 200 µL of sterile TSB. The plates were incubated at 30 °C for 24 h, and 5 μM of the cell permeant nucleic acid dye SYTO 9 (diluted 1:1000 in TSB from a SYTO 9 stock solution at 5 mM in DMSO; Invitrogen, France) were added to the 200 µL culture, obtain green fluorescent bacteria. For each strain, at least 9 to 15 wells were analyzed independently.

### 2.3. Macrocolony Experimental Conditions

For colony architectural formation, 3 μL of an overnight culture in TSB were inoculated on 1.5% Tryptone Soya Agar (TSA) with 40 μg/mL Congo Red (Sigma-Aldrich, St. Quentin Fallavier, France) and 20 μg/mL Coomassie Brilliant Blue (Sigma-Aldrich, St. Quentin Fallavier, France). Congo Red has been shown to bind extracellular matrix components and allows to compare the ability of different bacterial strains, including *B. subtilis*, which binds to amyloidic proteins [45,46]. The Coomassie Blue has a high affinity to bind proteins and is commonly used to detect, visualize, and quantify proteins separated on polyacrylamide gels [47,48]. The plates were then incubated at 30 °C for 6 days. Digital images of the colonies on the plates were taken using a Canon EOS 80D with 24 MP (6000 × 4000 pixels). Macrocolony experiments were performed three to five times independently.

### 2.4. Swarming Experiment Conditions

Cultures for the swarm inoculum were prepared in 10 mL B-medium inoculated with a single colony and shaken overnight at 37 °C. The culture was then diluted to an OD_600nm_ of approximately 0.1 and grown until it reached an OD_600nm_ of approximately 0.2. This procedure was repeated twice and finally the culture was grown to T4 (4 h after the transition from exponential growth). The OD_600nm_ was measured and the culture was diluted, and 2 μL of diluted bacterial culture (10^4^ CFU) were inoculated at the center of B-medium agar plate and incubated for 24 h at 30 °C with 50% relative humidity. Plates (9 cm diameter) containing 25 mL agar medium (0.7% agar) were prepared 1 h before inoculation and dried with lids open for 5 min before inoculation. Pictures were taken by a digital Nikon Coolpix P100 (10 MP) camera. Swarming experiments were repeated three to five times independently.

### 2.5. Pellicle Experiments

After an overnight culture in TSB at 30 °C, 10 μL of the bacterial suspension were used to inoculate 2 mL of TSB in 24-well plates (TPP, Trasadingen, Switzerland). Plates were then incubated at 30 °C for 24 h. Digital images of the pellicles were taken using a digital Nikon Coolpix P100 (10 MP) camera. This experiment was repeated three up to five times independently for each condition.

### 2.6. Non-Invasive Confocal Laser Scanning Microscopy (CLSM) of Submerged Biofilms

Immersed biofilms were observed using a Leica SP8 AOBS inverter confocal laser scanning microscope (CLSM, LEICA Microsystems, Wetzlar, Germany) at the INRAE MIMA2 platform (www6.jouy.inra.fr/mima2_eng/ accessed on 1 December 2020). For observation, strains were tagged fluorescently in green with SYTO 9 (1:1000 dilution in TSB), a nucleic acid marker. After 20 min of incubation in the dark at 30 °C to enable fluorescent labeling of the bacteria, plates were then mounted on the motorized stage of the confocal microscope. Biofilms on the bottom of the wells were scanned using a HC PL APO CS2 63x/1.2 water immersion objective lens. SYTO 9 excitation was performed at 488 nm with an argon laser, and the emitted fluorescence was recorded within the range 500–600 nm on hybrids detectors. The 3D (xyz) acquisitions were performed (512 × 512 pixels, pixel size 0.361 µm, 1 image every z = 1 µm with a scan speed of 600 Hz). Easy 3D projections were constructed from Z-series images using IMARIS v9.0 software (Bitplane AG, Zurich, Switzerland). Biofilms biomass was estimated through extraction of the biofilm biovolume (in µm^3^/µm^2^) after isosurfaces automatic detection using the IMARIS quantification module from a minimum of twenty confocal image z-series.

### 2.7. Statistical Analysis

One-way ANOVA was performed using GraphPad Prism 8 software (GraphPad, CA, USA). Significance was defined as a *p* value associated with a Fisher test value lower than 0.05.

## 3. Results and Discussion

### 3.1. Bacillus Subtilis NDmed forms Highly Structured Biofilms Compared to the NCIB3610 and 168 Strains

In the last decades, NCIB3610 has been widely used as a model for the “wild type” of *B. subtilis*. This strain has been shown to form more elaborate and robust biofilm communities when compared to the domesticated laboratory stain 168 [49,50]. However, in both the NCIB3610 and 168 strains, the *ypqP* gene is disrupted by the SPβ prophage, contrary to several sequenced natural isolates of *B. subtilis* [13]. This gene has been shown to be involved in the strong spatial organization of biofilms of the undomesticated *B. subtilis* NDmed strain, both at air and liquid interfaces [13]. In this study, a phenotypical characterization of NDmed grown under different laboratory culture conditions was performed, in comparison with the classical reference strains NCIB3610 and 168.

**Figure 1:**
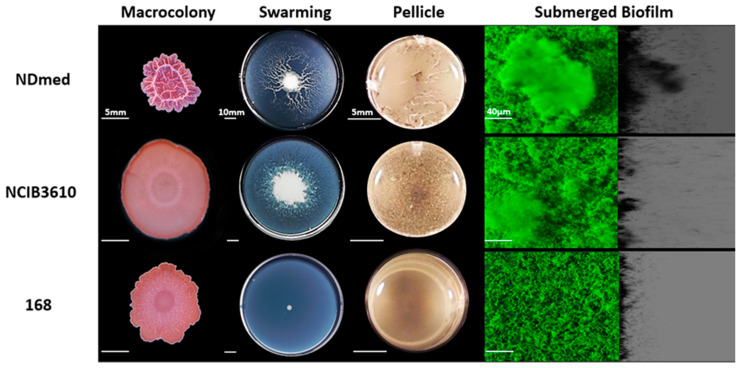
Comparative phenotype for *B. subtilis* strains on different laboratory assays. Macrocolonies grown on 1.5% TSA for 6 days at 30 °C after a central spot of 3 µL of an overnight bacterial culture in TSB (scale bars 5 mm). 0.7% of B-synthetic medium is used for swarming plates (9 cm diameter) that are incubated for 24 h at 30 °C (scale bars represent 10 mm). For pellicles, bacterial cells have been cultured in a 24-well plate with TSB for 24 h at 30 °C (scale bars 5 mm). Macrocolony, swarming, and pellicle images are representative for the majority of the phenotype from at least three replicates for each strain, which reveal variation for the surface architecture. In a 96 well microplate system, immersed biofilms are labeled by SYTO 9 after 24 h of incubation at 30 °C. The shadow on the right represents the virtual lateral shadow projection of the submerged biofilm (scale bars represent 40 µm).

Macrocolonies of these strains were observed after being grown for 6 days on indicator plates containing both Congo Red (labeling amyloidic proteinaceous compounds in *B. subtilis* biofilm matrix) and Coomassie blue (proteinaceous matrix counterstain) [46,47]. As shown in Figure 1, the NDmed strain formed a highly structured and more compact macrocolony, contrary to the NCIB3610 and 168 strains that formed flat macrocolonies without or with only very fine wrinkles. In addition, the NDmed macrocolony was more intensely stained by the Congo Red, indicating a higher amount of exopolymeric substances and proteins produced compared to the two other strains.

As the biofilms formed by these three strains had such profound architectural differences, we wondered whether they might also present marked differences in another structured multicellular behavior i.e., swarming. Hence, to better visualize differences between them, semi-solid plates (swarming plates) were used as a 2D model to view bacterial surface colonization initiating from a macrocolony. Dendritic swarming pattern of *B. subtilis* was previously best characterized on a synthetic fully defined medium (B-medium) with optimized nutrient and temperature conditions [28]. Figure 1 shows the swarming patterns obtained on the synthetic B-medium (0.7% agar) after 24 h of incubation at 30 °C for the studied *B. subtilis* strains. Obviously, both NDmed and NCIB3610 showed swarming on B-medium but with varied dendritic patterns. NCIB3610 displayed a thin highly complex dendritic swarming pattern that spread all over the plate within 24 h of incubation, whereas NDmed swarmed with a thick countable less spread dendritic pattern. The mother colony of the NDmed appears to be highly structured with slimy texture when disrupted mechanically by a loop. On the other hand, a less structured widely spread mother colony was formed by NCIB3610, suggesting that less extracellular polymeric substances are produced in this strain compared to the NDmed strain. The mother colony in a swarm for both NDmed and NCIB3610 closely resembles the structural architecture of the macrocolonies formed. Consistent with previous observation, the 168 *B. subtilis* strain failed to swarm on this synthetic medium, essentially because of a frameshift mutation in the *sfp* gene, required for the surfactin biosynthesis that facilitates the migration over the surface by reducing the surface tension [27].

Other models of biofilm are formed in liquid cultures, either at the air-liquid interface (pellicle) or as submerged biofilms at the solid-liquid interface [8,10,11,12]. To characterize the ability of *B. subtilis* to adhere and to form submerged multicellular communities on surface, CLSM has been used to acquire confocal stack images for the submerged biofilms, from which an Easy-3D reconstruction by the IMARIS software could reveal the three-dimensional structure with a lateral virtual shadow projection. As shown in Figure 1, and in accordance with previous reports, *B. subtilis* NDmed formed well-structured air-liquid biofilm (pellicle) and highly spatially organized submerged biofilm at the solid-liquid interface [11,13,30].

NCIB3610 strain did not form a thick pellicle within 24 h of incubation at 30 °C but produced well-structured biofilms (with a biovolume of 11 µm^3^/µm^2^, significantly smaller than the 14 µm^3^/µm^2^ biovolume formed by the NDmed, *p* < 0.05). The 168 strain, as previously been observed [11], was unable to form a pellicle in these conditions and displayed only a much less dense submerged biofilm (with a 6 µm^3^/µm^2^ biovolume) in comparison with the two other *B. subtilis* strains (*p* < 0.05).

In comparison between the three *B. subtilis* strains studied here, NDmed displayed complex architectural biofilm formation on/in both solid and liquid medium, and had the ability to swarm rather efficiently.

### 3.2. Mutants Affected in Matrix-Producing Components Fail to Form Well-Firmed Surface Cohesive Biofilms

In order to determine whether the genes involved in *B. subtilis* colony and pellicle formation also govern submerged biofilm formation, we constructed a set of derivative mutants of the NDmed strain and analyzed the corresponding phenotypes in the different biofilm models.

Extracellular matrix, mainly composed of polymeric substances, is essential for the biofilm structural formation. In *B. subtilis*, the amyloid-like fiber TasA encoded by the *tapA-sipW-tasA* operon, and the polysaccharides synthesized by the products of the *epsA-O* operon are mainly responsible for the synthesis of biofilm matrix, which bundles cells together and maintains their stability [2,46,49,51,52]. In addition, the BslA protein exhibits amphiphilic properties by forming a hydrophobic layer at the air interface [53] and activates the formation of complex colony development and pellicle formation [20,54]. Poly-γ-glutamate (γ-PGA), a secreted polymeric substance that accumulates in the culture media like the biofilm matrix [9] and in the capsule, is synthesized by the enzymes encoded by the *cap* operon. Recently it has been shown that in many tested environmental *B. subtilis* isolates γ-PGA production contributed to the complex morphology and robustness by enhancing cell-surface interactions of the colony biofilms [55]. The *ypqP* gene in both *B. subtilis* strains 168 and NCIB3610 is disrupted by the SPβ prophage, whose excision during sporulation phase reconstitutes a functional *ypqP* gene allowing addition of polysaccharides to the spore envelope [32]. In the undomesticated NDmed strain, *ypqP* non-disrupted by the SPβ prophage, has been identified as a requirement for the spatial biofilm organization [13].

**Figure 2:**
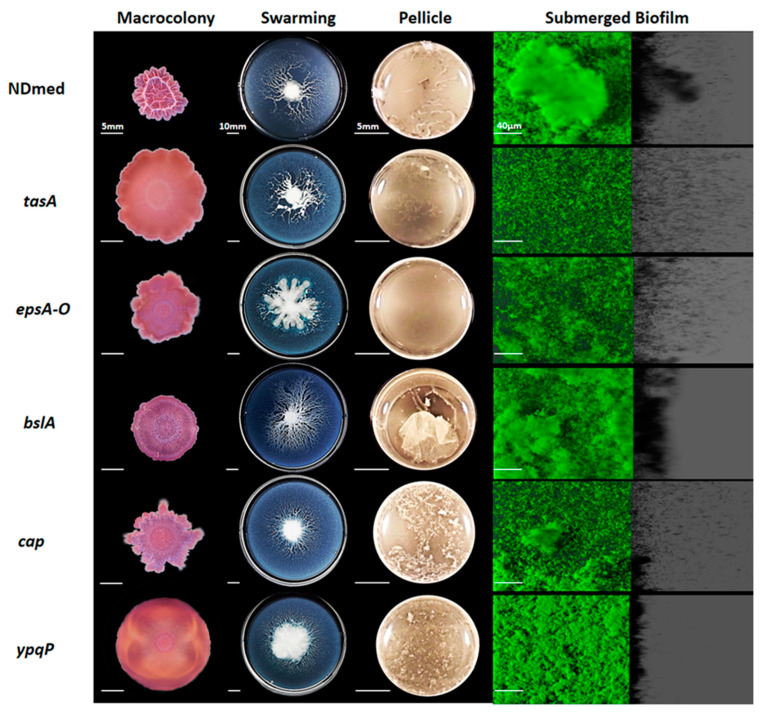
Different *B. subtilis* NDmed mutants of genes involved in extracellular matrix production on different culture assays. On 1.5% TSA, macrocolonies grown for 6 days at 30 °C after a central spot of 3 µL of an overnight bacterial culture in TSB. For swarming model, 2 µL of bacterial culture (10^4^ bacterial dilution) have been inoculated on the middle of 0.7% B-medium plates and cultured for 24 h at 30 °C. In a 24-well plate, bacteria in TSB are cultured at 30 °C and pellicles were obtained after 24 h. Macrocolony, swarming, and pellicle images are representative for the majority of the phenotype from at least three replicates for each strain revealing the effect of mutations on the biofilm formation. In a microplate system, immersed biofilms are labeled by SYTO 9 after 24 h on incubation at 30 °C. The shadow on the right represents the vertical projection of the submerged biofilm (scale bars represent 40 µm).

Figure 2 shows the effect of matrix gene mutation on different laboratory culture assays. Macrocolonies formed by *tasA*, *epsA-0*, *bslA*, *cap*, and *ypqP* mutants on TSA agar medium were flat contrary to the highly structured and wrinkled wild type NDmed colony (Figure 2). Interestingly, the *tasA* mutant was the least stained, by proteinaceous dyes, indicating a drastic negative effect of the corresponding mutation on extracellular matrix production.

Effects of matrix gene mutations on surface motility were visualized through swarming plates. All mutants affected in matrix synthesis tested were observed to swarm better than the wild type NDmed strain after 24 h of incubation on minimal B-medium. The mother colony (place of bacterial inoculation) for the *tasA*, *epsA-O*, and *ypqP* mutants was producing a very viscous and loose matrix. This suggests that all together the TasA (amyloid-like fibers) with the exopolysaccharide synthesized (through the products of *epsA-O* and *ypqP*) are important for the cell interlock and the structural stability in a biofilm.

However, it is difficult to differentiate the importance of each gene individually on the biofilm structural formation on agar. Hence, submerged biofilms revealed how in the *tasA* and *epsA-O* mutants biofilm cells were clearly unbundled and unable to form structured biofilms (Figure 2). Submerged biofilm formed by the *bslA* mutant was not affected at all, and those formed by the *cap* and the *ypqP* mutants were quite less affected after 24 h of incubation. Such observation has been numerically confirmed by an estimation of the biovolume and the thickness of submerged biofilms formed for all NDmed mutants studied here, which are represented in Figure 5. Indeed, in this study the *ypqP* mutation had a less effect on submerged biofilm biovolume and thickness after 24 h of incubation, however, the effect was more drastic when compared to the wild type NDmed after 48 h of incubation [13]. Moreover, *ypqP* was slightly expressed after 24 h and strongly transcribed only after 48 h (our unpublished data). This could suggest that *ypqP* is involved in the late structural biofilm spatial organization.

Regarding biofilms formed on liquid-air interface, our observations also highlight the importance of amyloid fibers and exopolysaccharides in the biofilm formation. In rich medium after 24 h of incubation the *tasA* and *epsA-O* mutants could form only very thin delicate pellicle (Figure 2), similar to what has been shown by previous studies on *B. subtilis* NCIB3610 [46,52]. As for the *ypqP* and *cap* mutants a less structured pellicle was formed. On the other hand, a delicate pellicle formed by the *bslA* mutant was very fragile and sensitive to any small plate movement, and sank to the bottom of the well due to cells lacking the hydrophobic layer that allows the pellicle to be stable at the air-liquid interface. These results suggest that *tasA* and *epsA-O* are crucial matrix genes, required in architectural settlement of *B. subtilis* multicellular communities in the different biofilm models. The genes *cap*, *ypqP*, and *bslA* also play an important role in formation of a highly structured and stable biofilm but in a more model-dependent way.

### 3.3. Motility and Autolysins are Essentially Required for Architectural Submerged Biofilm Formation of B. subtilis NDmed

In the mid-exponential growth phase of *B. subtilis*, two populations of cells were described: individual motile cells, and long chains of sessile cells [56]. Motility is a way for bacteria to colonize more favorable niches. Bacterial motility has also a positive role in nascent biofilm maturation and spreading, as it has been shown that motile cells can create transient pores that increase the nutrient flow in the matrix of mature biofilms [57]. In *B. subtilis,* flagellar motility studies have focused on both swarming over semi-solid agar plates and swimming in liquid culture [27,28,56,58]. As previously shown, *B. subtilis hag* mutants, affected in a gene encoding flagellin protein for flagellum formation, fail to swarm over different media tested including the B-medium [27,29]. In liquid culture, *B. subtilis hag* mutant was shown to have a delayed flagellar formation [10,58].

**Figure 3:**
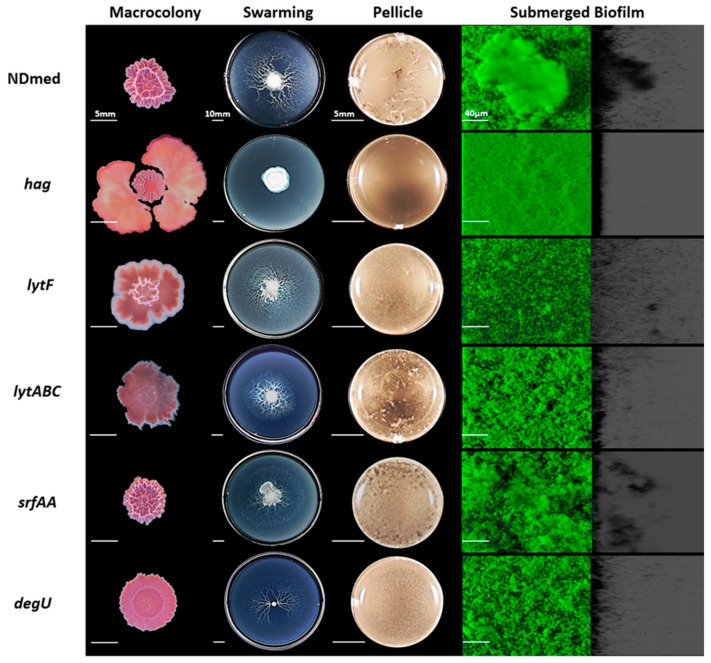
Motility and autolysin genes mutants of *B. subtilis* NDmed strain on different laboratory assays. Macrocolonies for mutated regulator genes are cultured on TSA for 6 days at 30 °C. Swarming plates are formed on B-synthetic medium (0.7% agar) that are cultured for 24 h at 30 °C. Pellicle and submerged biofilms were formed after 24 h of incubation at 30 °C in TSB medium. For submerged images the scale bars represent 40 µm. Macrocolony, swarming, and pellicle images are representative for the majority of the phenotype from at least three replicates for each strain revealing the effect of mutations on the biofilm formation.

In Figure 3, the NDmed *hag* mutant formed a slightly wrinkled macrocolony on agar plate, while it failed to swarm on an optimal semi-solid plate. In static liquid culture after 24 h of incubation, this *hag* mutant was able to produce non-structured submerged compact biofilm with diminished thickness unaffecting the biovolume at the solid-liquid interface (Figure 5). Nevertheless, the *hag* mutant did not form pellicle at the air-liquid interface after 24 h of incubation in a rich medium (TSB). This suggested that the inability to swim prevented the cells to reach the air-liquid interface and thus inhibited or caused a delay in the formation of a pellicle, as previously observed [10].

For efficient growth and motility, bacteria need to continuously divide and adapt the cell wall composition (peptidoglycan), thanks to the autolysin system in *B. subtilis.* Expression of two major autolysin genes, *lytF* and *lytC* involved in cell separation is controlled by sigma factor D that also directs the transcription of motility and chemotaxis genes [24,59]. We have studied the effect of *lytF* and *lytABC* mutation on the different assays of biofilm formation (Figure 3). The NDmed *lytF* mutants showed better aerial (macrocolony and pellicle) biofilm formation than the *lytABC* autolysin mutant, that formed flat and pale color macrocolony (due to the reduced autolytic enzymes produced). However, in submerged biofilm, the *lytF* mutant was more affected and showed reduced biovolume (Figure 5A; *p* < 0.05) while the biofilm biovolume of the *lytABC* mutant was only slightly decreased. To look at the effect on motility, we have tested these mutant strains on swarming plates. Similarly to previous observation with *B. subtilis* NCIB3610 strain [59] the *lytF* mutant was able to swarm better than the *lytABC* mutant, which led to the proposition that *lytF* is principally dedicated in cell separation and *lytC* is more involved in the proper flagellar function [59]. Hence, among the different autolysins, encoded by more than 35 genes encoding peptidoglycan hydrolases, inactivation of only one gene will have an impact on one of the biofilm models studied. However, absolute long chain cells phenotype could not be always seen, since different autolysins could replace each other [24,60].

Interestingly, the *srfAA* mutation, affecting surfactin production and competence, has no effect on the structural biofilms developed as macrocolonies, pellicle, and submerged one (Figure 3) when compared to the wild type *B. subtilis* NDmed (*p* > 0.05). On swarming plates, surfactin production reduces surface tension during bacterial surface translocation. The 168 strain, carrying a frame-shift mutation in *sfp*, fails to produce surfactin and is thus unable to migrate over the B-medium swarming plate [27,29]. Moreover, studies with the NCIB3610 *sfrAA* mutant have also shown its inability to swarm [61]. However, either 168 or NCIB3610 *srfAA* mutants, have been shown to regain the ability of swarming, when provided with exogenous surfactin [27,61]. Interestingly, in our study, the NDmed *srfAA* mutant, which lacks a surfactin ring, displayed a monolayer dendritic swarming pattern having migrated from a more viscous mother colony, suggesting that other extracellular proteases have been secreted to facilitate the translocation.

Previous studies have shown that mutation of *degU* affects transcription of more than 200 genes, which intervene in the genetic network activation for both flagellum and biofilm formation [54]. It has been demonstrated that different levels of DegU~P co-ordinates *B. subtilis* multicellular behavior i.e., low level of DegU~P activates swarming motility and complex architectural colony formation whereas high level of DegU~P inhibits swarming and complex colony formation and is mainly required for the activation of exoprotease production [54,62]. In *B. subtilis* NCIB3610, DegU targets two proteins that have been shown to be involved in biofilm formation, YuaB (BslA) and YvcA (a putative membrane-bound lipoprotein). However, for the *B. subtilis* ATCC6051 strain, highly genetically similar to NCIB3610 (they are both descending from the original Marburg strain [37]), YvcA has been shown to be required only for complex colony formation but not for pellicle formation [20,54,62]. Hence, multicellular communities differ from strain to strain, which highlights the interest to test *degU* mutation affecting the undomesticated strain NDmed and observe its effect over the different laboratory assays (Figure 3). Such *degU* mutation has a negative impact on the complex architectural macrocolony formed on agar surface and only slightly affects the biovolume formed by the submerged biofilm (Figure 5A, *p* > 0.05). A slight delay was observed in the swarming motility as well as for the pellicle formation indicating that a complex regulatory network, like phosphorylated Spo0A [20], intervenes to ensure a comparable biofilm formation.

### 3.4. Mutation of B. subtilis NDmed Biofilm Regulators do Not Have the Same Impact on All Biofilm Models

**Figure 4:**
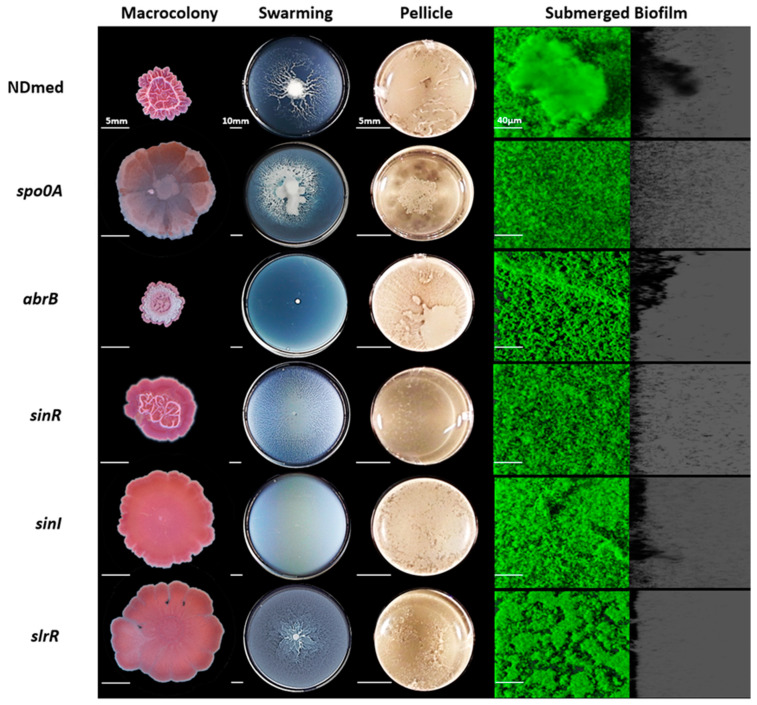
Mutational effect of global regulators required for biofilm development. Macrocolonies for mutants of regulator genes have been cultured on TSA for 6 days at 30 °C after a central spot of 3µl of an overnight bacterial culture in TSB. Swarming plates are formed by B-synthetic medium (0.7% agar) incubated for 24 h at 30 °C. Pellicle formed after 24 h of incubation at 30 °C in TSB medium. Macrocolony, swarming, and pellicle images are representative for the majority of the phenotype from at least three replicates for each strain revealing the effect of mutations on the biofilm formation. In a microplate system, immersed biofilms are labeled by SYTO 9 after 24 h on incubation at 30 °C. The shadow on the right represents the vertical projection of the submerged biofilm (scale bars represent 40 µm).

Spo0A, a key regulator of biofilm formation, is driven by exogenous and endogenous signals [63]. Activated Spo0A governs the genetic pathway controlling the matrix production gene expression by inducing SinI which binds and inhibits SinR, a repressor of the *eps* and *tapA-sipW-tasA* operons. Another role for Spo0A is to repress the expression of AbrB, a negative regulator for the initiation of biofilm formation [8,64]. Hence, the transition from surface-attached cells to three-dimensional biofilm structure is dependent on the activated Spo0A regulator [8]. To determine and clearly visualize the effect of these regulators on biofilm formation, *spo0A*, *abrB, sinR, sinI*, and *slrR* mutants of NDmed were tested under different biofilm culture conditions (Figure 4).

**Figure 5:**
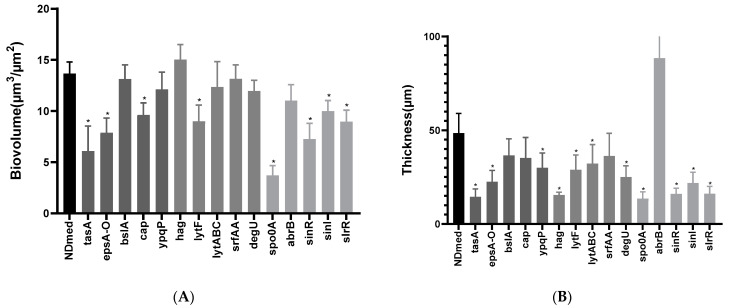
Submerged biofilms biovolumes and maximum thickness of the *B. subtilis* NDmed mutant strains studied. Biovolume (**A**) and maximum thickness (**B**) obtained were calculated from twenty confocal image series each. The color of the bars indicate gene categories (black for wild type NDmed, dark grey for matrix genes, grey for motility and autolytic genes, and light grey for global regulators). The error bars indicate the 95% confidence level, and the asterisk indicates the statistically significant differences (* is for *p* < 0.05) with the NDmed wild-type strain.

The *spo0A* mutant grew as a structureless spread macrocolony, while the *abrB* mutant showed a very vigorous and structured macrocolony on solid agar medium (Figure 4). In liquid culture, previous studies have shown that *B. subtilis spo0A* mutant cells were able to adhere to a surface and attach only as a monolayer form, suggesting that these mutants lack cell-cell interactions necessary for multicellular biofilm formation [8]. By using the CLSM, we could observe that the *spo0A* mutant cells did not form any thick submerged biofilm and rather remained essentially dispersed in the medium (Figure 4). These dispersed cells seemed to reach the surface of the liquid-air interface and form a highly disconnected pellicle-like structure in the middle of the well. On the other hand, the *abrB* mutant could form an extremely firm and highly structured pellicle, even more than that formed by the wild type NDmed strain, as well as thick highly structured architectural submerged biofilm (Figure 4 and Figure 5B). Quantification of the submerged biofilm biovolumes (Figure 5A) formed by the *spo0A* and *abrB* mutants assures the role of Spo0A/AbrB pair as the main regulator for biofilm formation. On swarming plates, the *abrB* mutant was strongly affected, where even though producing an extensive surfactin zone, it was only able to form few small bud-like structures that emerged from the mother colony and then failed to proceed further. A similar behavior was observed for the *abrB* mutant of *B. subtilis* (168 *sfp+*) whose cells within the bud accumulate as long-chain forms [29]. Besides this, we could observe that the *spo0A* mutant on the swarming plates (Figure 4) showed extensive motility that filled all the plate rapidly with viscous multicellular biofilm formation in the middle of the plate. This could indicate that this viscous layer is due to an extensive secretion of surfactin or of extracellular proteases from a huge number of bacterial cells that lack cell-cell interaction, facilitating the movement over the surface.

Biofilm formation, under appropriate conditions, is initiated by motile *B. subtilis* cells that adhere to the surface become sessile and form long chains of non-motile cells, held together by extracellular matrix. The transcription factor SinR, a central regulator in the assembly of *B. subtilis* cells into multicellular communities [17], controls both motility and biofilm formation by directly repressing the *eps* and *tapA-sipW-tasA* operons [65]. SinI, induced by phosphorylated Spo0A, binds directly to SinR and causes its inhibition. Moreover, SinI derepresses the action of SlrR [18,66]. SlrR, an additional regulatory protein, binds to and antagonizes SlrA, and thus constitutes a negative regulatory double loop with SinR, in which the *slrR* gene is repressed by SinR and in turn SlrR prevents SinR from repressing *slrR* [16,67]. SlrA could play only a minor role in biofilm formation; however, it can be substituted functionally by SinI, its equivalent paralog [16,18]. Hence, SinR is inhibited by two paralogous antirepressors, SinI and SlrA [16].

A *sinR* mutation, in the NCIB3610 strain has been shown to lead to the formation of extremely thick colony when compared to the wild type, while *sinI* or *slrR* mutants formed flat structureless colonies on agar surface [17,65]. We have investigated the role of these major gene regulators on submerged biofilm formation and motility in the *B. subtilis* NDmed strain. Figure 4 shows similar phenotype for both *sinI* and *slrR* mutants with flat structureless macrocolonies on agar surface; however, the *sinR* mutant formed wrinkled macrocolony less structured than that formed by the wild type.

Swarming is a phenomenon taking place in two consecutive stages, migration over the surface of highly motile cells followed by their differentiation to less motile matrix producing cells that become stacked in a three-dimensional structure [26,68]. On swarming plates and after 24 hr of incubation, the NDmed *sinR* mutant swarmed all over the plate with a multilayered biofilm dendritic pattern, which could indicate that swarming cells are unable to separate. In contrast, the NDmed *sinI* mutant eventually swarmed all over the plate in a monolayer form (Figure 4) similar to what has been described for *sinI* mutant in the NCIB3610 context [17]. This suggests that when matrix production genes are blocked, mutant bacterial strains were only able to reach the first stage of swarming. SlrR stimulates transcription of the *tapA-sipW-tasA* operon but not of the *eps* operon and represses genes that mediate cell separation [10,18]. Thus, *slrR* mutation affects the expression of TasA but not Eps production and promotes cell separation. On swarming plates, the NDmed *slrR* mutant was able to swarm rapidly in a monolayer form all over the plate with less structured biofilm in the mother colony (place of inoculation) when compared to the wild type (Figure 4).

In liquid culture, a NDmed *sinR* mutant cultivated in TSB medium for 24 h of incubation, formed very thin pellicle (Figure 4). This could be due to cells unable to reach easily the surface. The NDmed *sinI* mutant was able to form a rather good pellicle, suggesting that the motile swimmer cells were able to reach the surface. These phenotypes are similar to what has been observed previously for ATCC6051 *sinR* and *sinI* mutant strains [10,18]. A defect in flagellar formation in the *sinR* mutant [10,18] and a functional complementation between SinI and SlrA [16] in the *sinI* mutant could account for these phenotypes. Another hypothesis could be the occurrence of natural frameshift mutations within the *sinR* open reading frame, which suppress the blocking biofilm formation effects of a *sinI* mutation, as shown by Kearns *et al.* [17]. A NDmed *slrR* mutant could form a thin pellicle at the air-liquid interface, similarly to what has been observed in the NCIB3610 context [65].

The submerged biofilm biovolume of the NDmed *sinR* mutant (Figure 5A) was more negatively affected than that of the *sinI* or the *slrR* mutants when compared to the wild type NDmed (with a *p* < 0.05 for these three mutated genes compared to the wild type). This could stress the importance of motility and autolysin in the formation of biofilm and suggest that mutation in one gene could be overcome and controlled by other regulatory pathways. Thus, these results further indicate that the SinI/SinR pair are the main regulators controlling the mode of bacterial life, motile or sessile, cells.

## 4. Conclusions

Overall, this study highlights the value of the NDmed strain as an undomesticated, naturally competent *B. subtilis* isolate, to point out the effect of gene mutation on the different structural biofilm communities formed. Gene mutation could exhibit a similar impact on all the different biofilm models formed on different culturing conditions. For instance, the *tasA* and *epsA-O* gene mutation affected all the surface associated communities formed but improved surface translocation. However, the *bslA* gene mutation has a negative effect just on the aerial biofilm models, structural microcolonies, and the pellicle stability, and no effect on the submerged biofilm formation. Our results emphasize the importance of the submerged model to further understand the molecular mechanisms during biofilm formation. Biofilm development throughout different environmental culturing conditions could have similar genetic profile, but these multicellular communities can also display considerable differences on the structural, chemical, and biological heterogeneity levels across different biofilm models. A whole transcriptional analysis could be done for the differently localized heterogeneous compartments of a biofilm to further understand the core of the transcriptional network that takes place between and during the biofilm development.

## Data Availability

Not applicable.
